# Liposomal Incorporation to Improve Dissolution and Stability of Rosmarinic Acid and Carvacrol Extracted from Oregano (*O. onites* L.)

**DOI:** 10.1155/2018/6147315

**Published:** 2018-07-24

**Authors:** Juste Baranauskaite, Gülengül Duman, Gülcan Corapcıoğlu, Algirdas Baranauskas, Alpay Taralp, Liudas Ivanauskas, Jurga Bernatoniene

**Affiliations:** ^1^Department of Analytical and Toxicological Chemistry, Lithuanian University of Health Sciences, Medical Academy, Sukileliu pr. 13 LT-50162, Kaunas, Lithuania; ^2^Institute of Pharmaceutical Technologies, Medical Academy, Lithuanian University of Health Sciences, Eiveniu 4, LT-50161 Kaunas, Lithuania; ^3^Department of Pharmaceutical Technology, Faculty of Pharmacy, Yeditepe University Atasehir, İnönü Mah., Kayışdağı Cad., 34755, Istanbul, Turkey; ^4^Nanotechnology Research and Application Center, Sabanci University, Orta Mahalle, Üniversite Cad. No. 27, Orhanli, 34956, Tuzla, Istanbul, Turkey; ^5^Department of Drugs Technology and Social Pharmacy, Lithuanian University of Health Sciences, Medical Academy, Sukileliu pr. 13 LT-50162, Kaunas, Lithuania; ^6^Altınay Aerospace & Advanced Technologies Inc., Teknopark Istanbul No.1/4A, Pendik, Istanbul, 34906, Turkey

## Abstract

The potential antimicrobial benefit of high levels of rosmarinic acid (RA) and carvacrol (CA) in oregano (*O. onites* L.) extract has been limited until now by poor bioavailability arising from the low aqueous-phase solubility and slow dissolution behaviour of the lyophilized extract (E). To address this issue, various ratios of phospholipon 90H (P90H) and 1,2-dimyristoyl-*sn*-glycero-3-phospho-(1'-rac-glycerol), sodium salt (DMPG) were sonicated, yielding four empty liposomes (L1, L2, L3, and L90). After an initial selection process, Turkish oregano extract was internalized into the more promising candidates. Each empty liposome, extract-loaded liposome (LE1, LE2, and LE3), and freeze-dried control (E) was assessed in terms of structure, composition, RA and CA dissolution profile, storage stability, and, when relevant, zeta potential. Empty liposome L1, which was prepared using P90H and DMPG in a 1:1 ratio, displayed the most convenient encapsulation traits among the four unloaded types. Loaded liposome LE1, obtained by combining oregano extract and L1 in a 1:1 ratio, proved superior as a vehicle to deliver RA & CA when compared against control freeze-dried E and test liposomes LE2 and LE3. Dissolution profiles of the active compounds RA and CA in loaded liposomes were determined using a semi-automated dissolution tester. The basket method was applied using artificial gastric juice without pepsin (AGJ, 50rpm, 500mL). The pH value was maintained at 1.5 (37 ± 0.5°C). Aliquots (5ml) were manually extracted from parallel dissolution vessels at 1, 3, 5, 7, 10, 15, 20, 25, 30, 45, and 60-minute time points. Dissolution tests, run to completion on LE1, showed that approximately 99% of loaded CA and 88% of RA had been released. Shorter dissolution times were also noted in using LE1. In particular, the release profile of CA and RA had levelled off after only 25 minutes, respectively, depicting an impressive 3.0–3.3 and 2.3-2.6 rate increase compared to the freeze-dried control extract. The improved dispersibility of RA and CA in the form of LE1 was supported by particle size and zeta potential measurements of the liposome, yielding 234.3nm and −30.9mV, respectively. The polydispersity index value was 0.35, indicating a reasonable particle size distribution. To study storage stability, liposomes were stored (4°C, 6 months) in amber coloured glass containers (4 oz.). Each container held 30 capsules, which were stored according to the ICH guidelines prescribed for long-term storage (25°C ± 2°C; 60% ± 5% RH). Triplicate samples were withdrawn after 0, 3, 6, 9, and 12 months for analysis. Lastly, LE1 displayed good storage stability. The results imply that RA and CA can be conveniently and routinely delivered via oral and mucosal routes by first internalizing oregano extracts into appropriately engineered liposomes.

## 1. Introduction

Recent investigations continue to reveal the merit and benefit of utilizing natural products in human health issues. Indeed, the use of plants, parts of plants and isolated phytochemicals for the prevention, and treatment of various diseases have been practiced since ancient times. It is estimated that about 25% of the drugs prescribed worldwide ultimately originate from plants [1].

One natural compound of particular interest is an extract from oregano (*O. onites* L.), which contains high amounts of the antimicrobial compounds rosmarinic acid (RA) and carvacrol (CA) [2, 3]. Rosmarinic acid is the ester of caffeic acid and 3,4-dihydroxyphenyl lactic acid [4], while carvacrol is a monoterpenoid phenol [5]. In addition to antibacterial and antifungal activity, oregano essential oils also possess antioxidant, diaphoretic, carminative, antispasmodic, and analgesic activities [5, 6]. One isolated work has professed carvacrol as the compound mainly responsible for the biological activity of oregano [7].

The use of herbal remedies carries along problems, such as low solubility and hence limited absorption and bioavailability. Such biologically active compounds are also prone to* in vivo* hydrolysis, oxidation, and photolysis, urging the need for stabilization platforms [8, 9]. Technologies such as liquid crystal (LC) systems, polymeric and solid lipid nanoparticles (SLNs), precursors systems for liquid crystals (PSLCs), liposomes, and microemulsions have been reported to overcome such limitations [10]. Such drug delivery platforms improve compatibility, allowing substances with different physicochemical traits to be used within the same formulation. Some even make it possible to change the drug's apparent traits and hence behaviour in a biological environment [11].

Among the available breakthrough technologies, liposomes have somehow escaped attention in the deployment of oregano extracts. Liposomes depict spherical bilayer membranes, which are formed by combining one or more various amphipathic phospholipids, yielding nanovesicles (i.e., nanoliposomes) with an aqueous inner core, a hydrophilic inner and outer phosphate surface layer, and a hydrophobic lipid bilayer [12, 13]. Given these traits, liposomes can carry hydrophobic drugs, essential oils, herbal complexes, and moisturizers within the bilayer region [14, 15] as well as water-soluble materials within the aqueous core. Since the biologically active materials are harboured within the liposomal structure, liposome formulations can improve the stability, solubility, and bioavailability of bioactive compounds [16]. For these reasons, liposomes have been successfully utilized to encapsulate, deliver, and simultaneously release water-soluble, lipid-soluble, and amphiphilic materials [10]. Through encapsulation, liposomal carriers have successfully protected therapeutic agents from the extreme acidity found at the beginning of the gastrointestinal tract. Like other nanosized therapeutic agents, liposomal carriers also avoid first-pass clearance by diversion through the gut-associated lymphoid tissue (GALT), thus significantly improving the efficiency of oral drug delivery [17, 18]. As a final noteworthy point, liposomes are biocompatible and readily biodegradable.

In this study, the extract from Turkish oregano (*Origanum onites *L.) (hereafter termed oregano) was encapsulated into liposomes. The objective of this study was to exploit and optimize the carrier effect of liposomes in order to impart desirable features to the drug formulation, such as high encapsulation efficiency, rapid drug release, high release yields, and improved drug stability. To begin, initial work focused on preparing and physicochemically characterizing the optimal liposomal formulation in terms of particle size, polydispersity, zeta potential, and morphology. Subsequent work focused on determining the loadings of RA and CA and their* in vitro* release kinetics. Finally, liposomes and freeze-dried extracts were compared to ascertain the merit of liposomes as carriers for these poorly aqueous-soluble compounds.

## 2. Materials and Methods

### 2.1. Materials

Dried* Origanum onites *L. herb was obtained from “İnanTarım ECO DAB,” Turkey. Voucher specimens (No. L270711) are held by the Herbarium of the Department of Drug Technology and Social Pharmacy, Lithuanian University of Health Sciences. Ethanol (96%) as extraction solvent was purchased from Vilniaus degtinė (Vilnius, Lithuania). Phospholipon 90H (PL 90H) and 1,2-dimyristoyl-sn-glycero-3-phosphorylglycerol sodium salt (DMPG-Na) were graciously provided by Lipoid AG (Ludwigshafen, Germany). Purified water used for sample preparation and HPLC runs was produced using a Super Purity Water System (Millipore, USA). HPLC eluents: methanol (99.95%) was purchased from Carl Roth GmbH (Karlsruhe, Germany) and acetic acid (99.8%) from Sigma-Aldrich (St. Louis, MO, USA). Standards for HPLC analysis: carvacrol (>98%) was purchased from Sigma-Aldrich (St. Louis, MO, USA) and rosmarinic acid (>98%) from ChromaDex (Santa Ana, TX, USA).

### 2.2. Methods

#### 2.2.1. Preparation of Oregano Ethanol Extract

Oregano herb was ground in a IKA Works brand A11 model cross beater mill-type basic grinder (Guanghou, China) and sieved using a Retsch brand AS 200 model basic vibratory sieve shaker (UK) equipped with a sieve (125*µ*m). Powdered material (100g) was extracted with 90% (v/v) ethanol (1L) in a round bottom flask via the technique of heat-reflux extraction (95°C, 4h). The heat source was a Memmert brand WNB7 model water bath (Memmert GmbH & Co. KG, Schwabach, Germany). The recovered extract was filtered via vacuum. These conditions were previously deemed most convenient for extracting RA and CA from Turkish oregano [19].

#### 2.2.2. Preparation of Empty Liposome (L)

The liposome was prepared using a film technique as per the original method of Bangham [20]. Each phospholipid was dissolved using 2:1 (v/v) chloroform/methanol (30mL) in separate round-bottomed flasks. Phospholipid from these stocks was combined in various ratios (300mg phospholipid in total; see Table 1 for weight ratios). The solvents were removed (15 min, 25°C) using a Heidolph brand rotary evaporator (Schwabach, Germany). The thin film, which had formed along the inner wall of the flask, was dispersed in purified water with some shaking. The suspension was frozen and freeze-dried overnight (-80°C) using a Martin Christ brand Alpha 2-4 LD Plus model (Germany) lyophilizer. The procedure yielded empty liposomes L1, L2, L3, and L90.

Chloroform and methanol were used in the above procedure as opposed to nontoxic solvents and water for the simple reason that prior experience had routinely revealed best results when manipulating phospholipids. Rotary evaporation and lyophilisation served to rid the empty liposomes of virtually all of the undesirable solvent prior to engaging subsequent steps.

#### 2.2.3. Preparation of Extract-Loaded Liposome Formulations (LE)

To promote uniform particles and minimize clustering, empty liposomes were loaded with ethanolic* Origanum onites* L. extracts according to the thin film hydration method [21]. Variables included the phospholipid composition, the liposome-to-extract ratio, and the time and shear rate of the homogenization process. To prepare loaded liposomes LE90, LE2, LE1, and LE3, freeze-dried empty liposome L90 or L1 was dispersed into the ethanolic oregano extract at prescribed weight ratios (Table 1). The resultant mixture was homogenized (10k rpm, 5 min) using a Wise Tis HG-15D model homogenizer. Ethanol was removed under reduced pressure (15 min, 25°C) using a rotary evaporator (Heidolph, Schwabach, Germany) to form a thin film on the inner walls of the flask. The thin film was dispersed into purified water and freeze-dried overnight (-80°C) using a Martin Christ brand Alpha 2-4 LD Plus model freeze-dryer (Germany). L2 and L3 were not pursued in this study to encapsulate the drug, as L1 and L90 proved more convenient.

#### 2.2.4. Characterization of Liposome Formulations

Physical size traits of the liposome dispersions were assessed using a Malvern brand ZS 501 model Particle Sizer dynamic light scattering (DLS) instrument (Malvern, Worcestershire, UK); liposome dispersion (0.1mL) was diluted in purified water (1mL). The mean particle size, size distribution, and polydispersity index were expressed as an average of six trials. Zeta potential was also measured (25°C, 90° angle) using a Malvern brand ZS 501 model Zeta-sizer instrument (DLS). The results of the analysis were expressed as a mean value of 10 measurements.

#### 2.2.5. Scanning Electron Microscopy (SEM) of Liposome Dispersions

Scanning electron microscopy (SEM) (LEO SUPRA 35 VP) images of liposomes were acquired at two magnifications (3.5 & 10 kx) using a Zeiss brand electron gun (working distance 12.1mm and 8 mm; SE2 detection mode; beam voltage 10kV). To prepare samples for imaging, dry liposome dispersions were sputter coated using carbon.

### 2.3. Quantitative Analyses

#### 2.3.1. Powder Preparation for HPLC Analysis

To quantify RA and CA loadings, liposomes were accurately weighed (100mg) and dispersed in methanol (10mL). The contents were extracted (10 min) using an ultrasound bath (Memmert WNB7 water bath, Memmert GmbH & Co. KG, Schwabach, Germany). The extract was passed through a nitrocellulose filter (0.45*µ*m) and submitted to HPLC analysis.

HPLC analysis was carried out using a Waters brand 2695 model chromatography system (Milford, USA) equipped with a Waters brand 996 model PDA detector. Data were collected and analyzed using Empower 2 Chromatographic Manager System software (Waters Corporation, Milford, USA). Samples were chromatographed along a ACE 5 C18 250 × 4.6 mm column (Advanced Chromatography Technologies, Aberdeen, Scotland).

#### 2.3.2. HPLC Conditions to Determine RA

The dual solvent mobile phase was composed of solvent A (methanol) and solvent B (0.5 % (v/v) acetic acid in water). The following linear gradient elution profile was used: 95 % A/5 % B – 0 min, 40 % A/60 % B – 40 min, 10 % A/90 % B – 41 - 55 min, and 95 % A/5% B – 56 min. The flow rate was 1 mL/min and injection volume was 10 *μ*L. RA in the effluent was determined at a wavelength of 329nm. Quantification was carried out using the external standard method. A linear calibration curve was prepared (R^2^=0.999918), and the underlying peak areas permitted quantification [19].

#### 2.3.3. HPLC Conditions to Determine CA

A single mobile phase was composed of methanol and water (60/40, v/v). The flow rate was 0.6 mL/min and injection volume was 10 *μ*L. CA absorption was measured at 275 nm. The quantification was again carried out using the external standard method and associated calibration curve (R^2^=0.999) [19].

#### 2.3.4. Product Yield (%w/w) and Encapsulation Efficiency (%w/w)

Product yield was defined as the weight of liposomes after the process of internalization divided by the weight of liposome, which in theory should have been measured had encapsulation been %100 effective and material losses zero. Yields were calculated as

(1) $$\begin{array}{c} Yield\;\;\left(\;\%\;\right)=\frac{Dry\;\;weight\;\;of\;\;extract\;\;loaded\;\;liposomal\;\;nanoparticles\;\;formed\;\;after\;\;encapsulation}{Summed\;\;dry\;\;weight\;\;of\;\;empty\;\;liposome\;\;and\;\;extract\;\;added\;\;prior\;\;to\;\;encapsulation}\times 100 \end{array}$$

where the dry weight of empty liposome refers to the lyophilized 1:1 PL 90H/DMPG system, the dry weight of the loaded liposome refers to lyophilized extract-encapsulated liposome, and the dry weight of extract refers to residue in the ethanolic oregano extract following removal of solvent. The yield of freeze-dried extract E was defined differently in view of the absence of any liposomes. Here, the yield was simply the ratio of the dry weight of dispersed E divided by the total dry weight initially added to the dispersion medium.

Encapsulation efficiency (%) was defined differently so as to emphasize the bioactive material as opposed to the entire liposome [22]:

(2) %EE=weight  of  bioactive  compound  added  to  load  the  liposomes  –  weight  of  unincorporated  bioactive  compoundweight  of  bioactive  compound  added  to  load  the  liposomes×100%

where the bioactive compound is RA, CA, or both extract materials depending on how the quantification is defined and carried out.

Quantitative estimation of RA and CA in loaded liposomes was carried out using HPLC. Liposomal formulation (100mg) was suspended in a 1:1 (v/v) 96% ethanol / methanol cosolvent (10ml). The suspension was sonicated (10 min), filtered through a nitrocellulose membrane (0.22*μ*m), and analysed as described below.

#### 2.3.5. Preparation of Capsules Fillings

The composition of capsules is shown in Table 1. Capsules were filled using a Capsuline brand manual capsule filling machine (USA).

#### 2.3.6. Determination of Dissolution Profiles and Their Variability

Dissolution profiles of the active compounds RA and CA in loaded liposomes were determined using a SOTAX brand AT 7 smart model semiautomated dissolution tester (Switzerland). The basket method was applied using artificial gastric juice without pepsin (AGJ, 50rpm, 500mL). The pH value was maintained at 1.5 (37 ± 0.5°C). Aliquots (5ml) were manually extracted from parallel dissolution vessels at 1, 3, 5, 7, 10, 15, 20, 25, 30, 45, and 60 minute time points, filtered through a nitrocellulose membrane (0.45*µ*m), and quantified via HPLC. The dissolution media in each vessel was topped off with fresh dissolution fluid (5mL) to restore the original volume. The mean value of six trial runs and a standard deviation for each type of nanoparticle were calculated. The evaluation of dissolution profiles was carried out in triplicate.

#### 2.3.7. Evaluation of Stability

To study storage stability, liposomes were stored (4°C, 6 months) in amber coloured glass containers (4 oz.). Each container held 30 capsules, which were stored according to the ICH guidelines prescribed for long-term storage (25°C ± 2°C; 60% ± 5% RH). Triplicate samples were withdrawn after 0, 3, 6, 9, and 12 months for analysis. Parameters were assessed such as particle size, zeta potential, PDI, %EE of CA & RA, organoleptic state, average weight, and drug content.

### 2.4. Statistical Analysis

Raw data was assessed using ANOVA statistical testing (specifically one-way analysis of variance) and Tukey's multiple comparison test. For this purpose, a software package was utilized (Prism v. 5.04, GraphPad Software Inc., La Jolla, CA, USA) with statistical significance being defined as p < 0.05.

## 3. Results and Discussion

### 3.1. Preparation and Characterization of Liposome

In commencing this work, initial emphasis had been placed on preferentially preparing zwitterionic liposomes comprised of P90H, the reason being that biological surfactants can be arranged in decreasing order of toxicity according to the commonly observed trend for oral and parenteral applications: cationic > anionic > amphoteric ≥ nonionic [23–27]. When initial testing revealed inconveniences in using only P90H, a combination of P90H and anionic DMPG was introduced, with emphasis being placed on using the minimal amount of DMPG needed to resolve the inconvenience. Following more iteration, a 1:1 P90H/DMPG ratio (L1) proved itself to be the liposome of choice for conducting subsequent extract-encapsulation studies, as this liposome displayed favorable mean particle size, zeta potential, and PDI traits. Table 1 is a formulation summary of some of the finalized liposome-based candidates used in this study. The subsequent internalization of* Origanum onites *L. was successfully carried out via the method of Fricker et al. using empty liposomes L90 and L1 to yield extract-loaded liposomes LE90, LE1, LE2, and LE3. Several iterations were necessary and minor protocol variations were implemented to arrive at cluster-free, reasonably uniform particles and a good level of extract internalization [21].

Summarized by Table 2, standard methods were used in this study to ascertain the particle size, polydispersity index (PDI), and zeta potential of (i) empty (L90) and extract-loaded (LE90) liposomes prepared from P90H, and (ii) empty (L1) and extract-loaded (LE1, LE2, LE3) liposomes prepared from 1:1 (wt/wt) P90H/DMPG (*vide*Table 1 for more information regarding composition).

The mean particle size displayed considerable variation in response to the liposome composition and extract loading. Among the empty liposomes, L90, which comprised P90H, had the largest particles. The particle size of empty liposomes was much smaller for P90H/DMPG combinations and the average diameter varied with the phospholipid ratio. The highest mean particle size for empty P90H/DMPG composite liposomes was L1. L1 was in fact two times larger on average than L3 (Table 2). The largest mean particle size (2660 ± 17.02 nm) was obtained in the case of LE90, whereas the smallest mean particle size corresponded to LE1 (234.3 ± 35.56 nm).

The polydispersity index (PDI) value is important in that it shows the size distribution of the liposomes, which can correlate to stability. A PDI value of 1.0 indicates a very broad size distribution or presence of large particles or aggregates, which could sediment. An optimum PDI value is 0.30 or less, signifying that 66.7% of all nanovesicles are the same size [28]. The PDI values obtained in this work ranged from 0.2 to 0.65 (Table 2), showing high-to-medium homogeneity of the liposome particle sizes. The highest PDI value was observed over the course of dispersing the freeze-dried extract. PDI values for LE90 and LE1 were 0.33±0.06 and 0.35±0.02, respectively. The PDI values of LE1 and LE90 were more or less the same. The PDI generally revealed a respectable but occasionally overly excessive size distribution in selected empty liposomes. The value ranged from 0.23±0.02 for L90 to 0.54±0.02 for L3. The highest PDI value was observed in the freeze-dried extract, indicating a heterogeneous system. Such a finding was not surprising, as the bulk process of freeze-drying would be anticipated to seed substantial particle size heterogenity compared to nanoparticle systems such as liposomes. The approximate PDI values for LE90 (0.33) and LE1 (0.35) indicated a relatively homogeneous size distribution. The differing phospholipid compositions in L90 and L1 did not affect the size distribution value to a significant degree, which is in agreement with other studies [29].

The zeta potential is an important parameter used to evaluate the dispersional stability of liposomal formulations. It characterizes the particles surface charge and gives an indication about repulsive forces between particles, thus allowing one to predict the stability of dispersions [29]. As a general rule of thumb, zeta potential values of < -30mV and > 30mV create stable systems because the high surface charges induces repulsion, thereby preventing aggregation [16]. Smaller absolute zeta values may also suffice if the electrical double layer thickness is comparable to or greater than the particle size. In this work, the zeta potential varied between -49.5±2.12mV for LE3 and 12.45±7.47mV for LE90 (Table 2). These variations corresponded to differences in lipid ratio of the various liposome formulations. Potentials ranged from -40.5±2.08mV for L2 to +1.53±0.14mV for L90. The relatively positive zeta potential of LE90 was not surprising in that the liposome was deficient in anionic surfactant. It also followed to reason that the small absolute zeta potential of LE90 had prompted coalescence and large average particle sizes (just as in the case of LE2). Given that the composite phospholipid liposomes (empty and loaded) had negative surface charges, the difference could be attributed to the presence of anionic DMPG. It is unclear as to what extent the carboxylic acid of RA contributes to the zeta potential; however, given that the zeta potential in transcending from L90 to LE90 was still positive, it can be assumed that RA's contribution is minor compared to DMPG. This assessment potentially conflicts with previous work in which the oregano herb is professed to be a major contributor of the overall zeta potential [30]. Subtle trait differences between the model systems of the current work and cited work could likely explain the conflicting observation. All in all, L1, L2, LE1, and LE3 all yielded substantially negative zeta potentials and small particle sizes, indicative of good stability against aggregation. Such results are consistent with another group [31].

**Figure 1:**
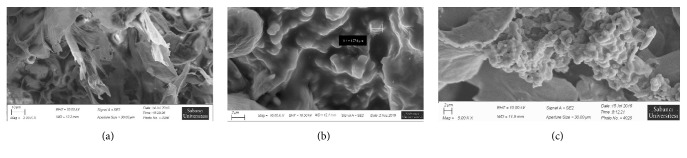
SEM micrographs of freeze-dried Oregano extract E (a) liposome vehicles L1, (b) liposome LE1, and (c) formulations.

SEM is the most convenient visual technique to probe the mean size and the surface morphology of particles [24]. The morphology of oregano extract-loaded LE1, freeze-dried E, and empty liposome L1 are shown in Figure 1. SEM imagery showed that most particles in LE1 were near-spherical and uniform in size with smooth surfaces. In cases of agglomeration, the agglomerate sizes ranged up to 1 *μ*m. In the case of LE1, agglomerates were still composed of small particles in the nanometer range. The arrangement of the nanoparticles formed an apparent porous network. L1 particles were spherical and uniform in shape and somewhat smaller than LE1. SEM also confirmed that the anionic lipid contribution in L1 appeared to yield better shaped particles with less interparticle interactions; such an observation would support the mean particle size differences noted between L90 and L1. Incorporation of oregano extract into L1 did not appear to have caused any morphological changes or crystal formation. Morphological analysis of the freeze-dried extract powder revealed the absence of small spherical granules, which implied poor dispersibility and hence low bioavailability.

### 3.2. Product Yield and Encapsulation Efficiency Determination

**Figure 2:**
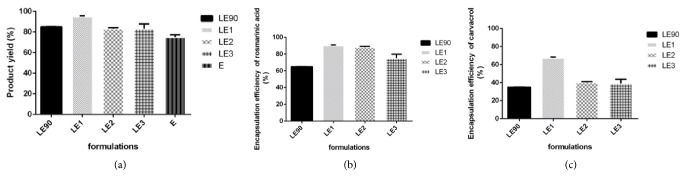
Product yield and encapsulation efficiency of various liposome formulations.

As shown in Figure 2, the best yield corresponded to LE1, which was approximately 1.2 times greater than E, LE90, LE2, and LE3 (given p<0.05). In terms of yield, there was no significant difference between LE90 and LE3 (p<0.05). Interestingly, higher extract to liposome ratios (LE2, LE3) prompted lower yields. The lowest product yield corresponded to freeze-dried E (*vide* method section for further discussion).

The encapsulation efficiency of RA and CA was determined independently of each other (*vide *methods section). As illustrated in Figure 2, efficiencies varied between 65 ± 0.78 % and 90 ± 1.2 % in the case of RA. The highest EE for RA appeared to correspond to LE1, however, there was no statistically significant difference in EE between LE1 and LE2 (given p<0.05). The lowest EE clearly corresponded to LE90. In the case of CA, EE's varied between 40.23±0.35% and 67.4±0.56%. The highest EE for CA again corresponded to LE1 (p<0.05) and this time encapsulation was clearly less efficient in the case of LE2. The same trend was noted for CA in the sense that LE90 fared poorest. As a general observation, the encapsulation efficiency varied according to the liposome type and size. It is generally accepted that the encapsulation efficiency of the active substances within liposomal structure can be affected by the size and/or specific surface area of liposome [32–35].

The highest encapsulation efficiency of RA & CA was observed in LE1, a 1:1 mixture of zwitterionic and anionic surfactant. It is unclear why better encapsulation of the carboxylic acid-bearing RA was not noted in LE90, given the lack of any anionic surfactant in the L90 liposomal structure. Still, RA does feature unusual behaviour, which might serve as a clue. In particular, other studies conducted on RA showed that RA could collapse the fluid space of bilayers through formation of interlayer bridges [16, 36]. Similar results were reported in the case of pistachio green hull ethanolic extract-loaded liposomes [16] and other similarities were cited in previous studies, which reported the high encapsulation efficiency of liposomes [29, 37, 38]. The lower EE for CA could be rationalized on the basis of its greater hydrophobicity compared to RA. It is conceivable that CA loads only in the lipid bilayer, which depicts a smaller volume space compared to the aqueous core of the liposome.

### 3.3. In Vitro Drug Release

**Figure 3:**
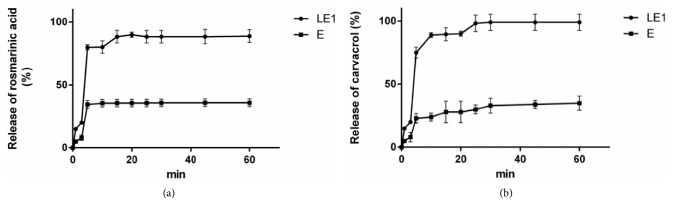
(a)* In vitro* release of CA from LE1 and E. (b)* In vitro *release of RA from LE 2 and E.

Figures 3(a) and 3(b) illustrate the* in vitro* dissolution profiles of LE1 and freeze-dried extract E. CA in LE1 dispersed into the solvent 3.0–3.3 fold more compared to E (Figure 3(a)). The data showed that LE1 had released 75% of its CA after only 5 minutes. Similar results were obtained previously [33, 39]. The same trend was observed with RA in the sense that LE1 prompted a 2.3–2.6 fold greater solution-phase concentration of dispersed RA compared to the control sample E (Figure 3(b)). Again, LE1 had released 80% of its RA content after only 5 minutes. In summary, LE1 displayed a significantly better* in vitro* dissolution profile and dissolution rate compared to the freeze-dried control sample E. Also noteworthy is that the release of CA from LE1 had approached 100% after 25 minutes. The improved release traits appeared to have rested on the liposome's ability to enhance the solubility or at least the dispersibility of hydrophobic and hydrophilic active compounds [29]. Such a claim is not without precedent; it has already been noted that the higher apparent dissolution and rate of dissolution of drugs in liposome formulations show promise as phospholipid carriers for enhancing the* in vivo* availability of active compounds [38, 40].

### 3.4. Release Kinetics

To better elucidate the release kinetics of CA & RA, the drug release versus time profiles of LE1 and E were shape-fitted against zero-order and first-order kinetic models (Tables 3(a) and 3(b)). The best correlation corresponded to the first-order model, implying the dependence of rate versus the concentrations of CA & RA. Such findings may also have reflected a pseudo first-order model, which escaped detection by typical kinetic analysis. Irrespective of whether or not the kinetics herein are first-order or pseudo first-order, the end result implies that the drug release rate will slow in time as the concentration of CA & RA diminishes.

Had zero-order kinetics been noted, certain benefits could have been claimed for this liposome-based technology. For instance, constant time-release kinetics could have imparted an additional element of convenience to drug delivery in medicine and agriculture. As it stands, the findings in Tables 3(a) and 3(b) imply that LE1 could serve as reservoir system for the continuous delivery of encapsulated CA and RA. However, the release kinetics will not be constant over time and must be monitored.

### 3.5. Storage Stability

Freeze-dried control extract sample E proved relatively hygroscopic in view that its moisture content had increased 1.4-fold (p<0.05) during the 28 days of stability testing in comparison to freshly prepared samples of E. Testing of E was terminated at this point because of the high moisture content.

Compared to control sample E, liposome LE1 was not hygroscopic. Hence, the stability of LE1 could be tested over a much longer storage period of 12 months at two different temperatures (4°C & 25°C ± 2°C; 60% ± 5% RH) (Table 4). In all aspects of testing, LE1 appeared physically, chemically, mechanically, and organoleptically stable. The mean size, zeta potential, PDI and EE of RA & CA were all comparable at the various time points. The disintegration times of the capsules were also independent of the storage period. With respect to mean particle size, only a 2.38% size increase (i.e., final size 240.3 ± 30.56 nm) was noted over the 12-month incubation period at 25°C ± 2°C and 60% ± 5% RH. In fact, particle size showed no statistically significant difference during the first 4 months. The same trend was noted at 4°C. PDI values also varied little during incubation at both temperature conditions, implying good stability. Indeed, no statistically significant difference was observed after the first month of storage (p >0.05). Paralleling the above was also the EE of RA & CA. The EEs were rock stable during the first 6 months of storage under both storage conditions. After 9 months, the EE of RA had diminished by only 3% compared to the zero time point. The EE of CA had diminished to a lesser degree than RA, implying near-perfect stability. Previous work has shown that stability can be challenged by long-term oxidation [11] so it appears that the liposomes of the current work are resistant to some extent. The slightly higher loss in EE of RA compared to CA could be related to RA's higher water-solubility; constant partitioning of RA into the aqueous core could conceivably have led to hydrolytic degradation. The same tendency has been reported for allicin encapsulated in liposomes; EE's had diminished over the course of 50 days of storage at 4°C [35].

## 4. Conclusions

In this study, various liposomal formulations were prepared with and without oregano extract. On the basis of physicochemical, morphological, and stability analyses, LE1 represented the best candidate for implementation as a therapeutic delivery agent. In terms of mean particle size, LE1 was appropriately sized to show good biological efficacy and about 4-6 times smaller than the other formulations. Moreover, the PDI value of LE1 indicated a sufficiently homogeneously disperse collection of particles. LE1 was a stable system, with a zeta potential at a sufficiently negative value. SEM imagery of LE1 visually supported the above claims in that it showed relatively homogeneously-sized nanospheres. In terms of the* in vitro* release profile, RA and CA incorporation into L1 liposomes dramatically improved the dispersion/dissolution traits of RA and CA compared to the freeze-dried control extract E. Without question, correlation studies indicated that the release kinetics of RA and CA proceeded via an apparent first-order model. Lastly, LE1 was remarkably stable over a 12-month storage period even under conditions of substantial humidity. These findings indicate that a liposomal oregano extract approach can improve the dispersibility and dissolution rate of RA and CA, thus implying a potentially promising alternative technology for the oral and mucosal application of such therapeutics.

## Figures and Tables

**Table 1:** Empty liposome (L), Oregano extract (E), and loaded liposome data: composition, weight ratio, mass and volume information, and formulation codes.

**Formulation**	**Composition**	**Ratio** ^**a**^	**Weight (mg, ml)**	**Code**
Liposome	P 90H*∗*	-	300mg	L90
Liposome	P 90H:DMPG*∗∗*	1:1		L1
Liposome	P 90H:DMPG	1:2		L2
Liposome	P 90H:DMPG	1:3		L3
Ethanolic *Oregano* herb extract	E		200ml	E
Liposome formulation	L90:E	1:1		LE90
Liposome formulation	L1:E	1:1		LE1
Liposome formulation	L1:E	1:2		LE2
Liposome formulation	L1:E	1:3		LE3

^a^Phospholipid ratio for L series, liposome/extract ratio for LE series.

*∗*P90H (Phosphatidylcholine).

*∗∗* DMPG (Na DMPG; dimyristoyl phosphatidylglycerol, sodium salt).

**Table 2:** Characteristics of various empty and Oregano extract-loaded liposomes.

**Formulation**	**Mean Size (nm)**	**Zeta potential (mV)**	**PDI (polydispersity index)**
L90	1176±148.6++	1.53± 0.14++	0.23±0.02⧫
L1	107.4±0.45	-35.7± 1.08	0.25±0.04 ⧫
L2	97.48±0.23	-40.5± 2.08	0.34±0.02⧫
L3	43.9±0.45	-49.5±4.08	0.54±0.02⧫
E	103.7±23.02	-23.7±7.47	0.65±0.03
LE90	2660±17.02	12.45±7.47 ++	0.33±0.06 ⧫
LE1	234.3±35.56 +	-30.9±9.12	0.35±0.02 ⧫
LE2	266.1±17.02 +	-28.5±0.47 +	0.47±0.06
LE3	634.3±35.56 +	-25.9±2.12 +	0.57±0.02 ⧫

*∗*RA: rosmarinic acid.

*∗∗*CA: carvacrol.

+ p ≤ 0.05 vs L2, L1, and L3.

++ p ≤ 0.05 vs L1, L3, L2, E, LE1, LE2, and LE3.

⧫ p ≤ 0.05 vs E.

**Table:** (a) Kinetic analysis of RA release profiles of LE1 and E

**Kinetic Model**	**LE1**	**E**
Zero-order		

R	0.8918	0.6909
R^2^	0.8086	0.6033

First-order		

R	0.9993	0.8609
R^2^	0.9987	0.8033

**Table:** (b) Kinetic analysis of CA release profiles of LE1 and E

**Kinetic Model**	**LE1**	**E**
Zero-order		

R	0.9018	0.6709
R^2^	0.8186	0.6233

First-order		

R	0.9903	0.7609
R^2^	0.9907	0.7033

**Table 4:** LE1 storage stability data under different conditions (4°C and 25°C).

**Conditions**	**Time (Mon)**	**Mean Size (nm)**	**Zeta potential (mV)**	**PDI**	**EE RA**	**EE CA**
***4***°***C±0.5***°***C/60***%*** ± 5***%*** RH***^***1***^	*Initial*	234.3±35.56	-30.9 ± 9.12	0.35±0.02	90.12±0.13	65.5±1.2

	1	236.9±12.5	-31.0±1.2	0.34±0.03	90±1.2	65.5±3.4
	3	234.1±23.12	-33.8±4.6	0.33±0.02	89.9±2.3	65.4±1.4
	6	240.1± 23.5	-32.78±3.9	0.33±0.01	89.9±1.3	65.3±0.3

***25***°***C±2***°***C/60***%*** ± 5***%*** RH***^***1***^	*Initial*	234.56±12.5	-32.6 ± 9.12	0.32±0.01	90.15±0.45	66.2±1.6

	1	235.01±16.7	-33.8±7.45	0.31±0.01	90.1±0.34	66±1.9
	3	236.7±12.6	-32.0±2.4	0.3±0.02	89.1±1.2	65.9±2.4
	6	239.98±9.4	-33.9±2.3	0.32±0.01	89.9±1.2	65.8±3.5
	9	239.99±8.2	-33.1±4.7	0.31±0.02	87.7±3.4	64.9±2.4
	12	240.0±2.4	-33.0±5.6	0.33±0.01	87.9±5.6	64.7±5.6

All values are mean values of triplicate samples of each time point.

^1^RH: relative humidity.

## Data Availability

The data used to support the findings of this study are available from the corresponding author upon request.

## References

[1] Patwardhan B., Partwardhan A. (2005). Traditional Medicine: Modern Approach for affordable global. *Traditional Medicine: Modern Approach for affordable global health*.

[2] Govaris A., Solomakos N., Pexara A., Chatzopoulou P. S. (2010). The antimicrobial effect of oregano essential oil, nisin and their combination against Salmonella Enteritidis in minced sheep meat during refrigerated storage. *International Journal of Food Microbiology*.

[3] Figiel A., Szumny A., Gutiérrez-Ortíz A., Carbonell-Barrachina Á. A. (2010). Composition of oregano essential oil (*Origanum vulgare*) as affected by drying method. *Journal of Food Engineering*.

[4] Petersen M., Simmonds M. S. J. (2003). Rosmarinic acid. *Phytochemistry*.

[5] Baranauskaite J., Ivanauskas L., Masteikova R., Kopustinskiene D., Baranauskas A., Bernatoniene J. (2017). Formulation and characterization of Turkish oregano microcapsules prepared by spray-drying technology. *Pharmaceutical Development and Technology*.

[6] Shafiee-Hajiabad M., Novak J., Honermeier B. (2016). Content and composition of essential oil of four Origanum vulgare L. Accessions under reduced and normal light intensity conditions. *Journal of Applied Botany and Food Quality*.

[7] Baser K. H. C. (2008). Biological and pharmacological activities of carvacrol and carvacrol bearing essential oils. *Current Pharmaceutical Design*.

[8] Kamel K. M., Khalil I. A., Rateb M. E., Elgendy H., Elhawary S. (2017). Chitosan-Coated Cinnamon/Oregano-Loaded Solid Lipid Nanoparticles to Augment 5-Fluorouracil Cytotoxicity for Colorectal Cancer: Extract Standardization, Nanoparticle Optimization, and Cytotoxicity Evaluation. *Journal of Agricultural and Food Chemistry*.

[9] Alexander A., Ajazuddin, Patel R. J., Saraf S., Saraf S. (2016). Recent expansion of pharmaceutical nanotechnologies and targeting strategies in the field of phytopharmaceuticals for the delivery of herbal extracts and bioactives. *Journal of Controlled Release*.

[10] Pinilla C. M. B., Noreña C. P. Z., Brandelli A. (2017). Development and characterization of phosphatidylcholine nanovesicles, containing garlic extract, with antilisterial activity in milk. *Food Chemistry*.

[11] Bonifácio B. V., da Silva P. B., Ramos M. A. D. S., Negri K. M. N., Bauab T. M., Chorilli M. (2014). Nanotechnology-based drug delivery systems and herbal medicines: a review. *International Journal of Nanomedicine*.

[12] Fan R., Gan L., Liu M. (2011). An interaction of helicid with liposome biomembrane. *Applied Surface Science*.

[13] Ozer A. Y. (2007). Alternative applications for drug delivery: Nasal and pulmonary routes. *Nanomaterials and Nanosystems for Biomedical Applications*.

[14] Lasic D. D. (1995). Applications of liposomes. *Handbook of Biological Physics*.

[15] Duman G., Aslan I., Özer A. Y., Inanç I., Taralp A. (2014). Liposome, gel and lipogelosome formulations containing sodium hyaluronate. *Journal of Liposome Research*.

[16] Rafiee Z., Barzegar M., Sahari M. A., Maherani B. (2017). Nanoliposomal carriers for improvement the bioavailability of high – valued phenolic compounds of pistachio green hull extract. *Food Chemistry*.

[17] Nguyen T. X., Huang L., Gauthier M., Yang G., Wang Q. (2016). Recent advances in liposome surface modification for oral drug delivery. *Nanomedicine*.

[18] Parmentier J., Hofhaus G., Thomas S. (2014). Improved oral bioavailability of human growth hormone by a combination of liposomes containing bio-enhancers and tetraether lipids and omeprazole. *Journal of Pharmaceutical Sciences*.

[19] Baranauskaite J., Jakštas V., Ivanauskas L. (2016). Optimization of carvacrol, rosmarinic, oleanolic and ursolic acid extraction from oregano herbs (Origanum onites L., Origanum vulgare spp. hirtum and Origanum vulgare L.). *Natural Product Research (Formerly Natural Product Letters)*.

[20] Bangham A. D., Standish M. M., Watkins J. C. (1965). Diffusion of univalent ions across the lamellae of swollen phospholipids. *Journal of Molecular Biology*.

[21] Fricker G., Kromp T., Wendel A. (2010). Phospholipids and lipid-based formulations in oral drug delivery. *Pharmaceutical Research*.

[22] Panda A., Meena J., Katara R., Majumdar D. K. (2016). Formulation and characterization of clozapine and risperidone co-entrapped spray-dried PLGA nanoparticles. *Pharmaceutical Development and Technology*.

[23] McClements D. J., Rao J. (2011). Food-grade nanoemulsions: formulation, fabrication, properties, performance, biological fate, and potential toxicity. *Critical Reviews in Food Science and Nutrition*.

[24] Ekelund K., Östh K., Påhlstorp C., Björk E., Ulvenlund S., Johansson F. (2005). Correlation between epithelial toxicity and surfactant structure as derived from the effects of polyethyleneoxide surfactants on Caco-2 cell monolayers and pig nasal mucosa. *Journal of Pharmaceutical Sciences*.

[25] van Hoogdalem E. J., de Boer A. G., Breimer D. D. (1989). Intestinal drug absorption enhancement: An overview. *Pharmacology & Therapeutics*.

[26] Liu R. R., Forrest M. L., Kwon G. S. (2008). *13 Micellization and Drug Solubility Enhancement Part II: Polymeric Micelles*.

[27] Torchilin V. P. (2006). *Nanoparticulates as Drug Carriers*.

[28] Sebaaly C., Charcosset C., Stainmesse S., Fessi H., Greige-Gerges H. (2016). Clove essential oil-in-cyclodextrin-in-liposomes in the aqueous and lyophilized states: From laboratory to large scale using a membrane contactor. *Carbohydrate Polymers*.

[29] Gharib R., Auezova L., Charcosset C., Greige-Gerges H. (2017). Drug-in-cyclodextrin-in-liposomes as a carrier system for volatile essential oil components: Application to anethole. *Food Chemistry*.

[30] Gibis M., Rahn N., Weiss J. (2013). Physical and oxidative stability of uncoated and chitosan-coated liposomes containing grape seed extract. *Pharmaceutics*.

[31] Rashidinejad A., Birch E. J., Sun-Waterhouse D., Everett D. W. (2014). Delivery of green tea catechin and epigallocatechin gallate in liposomes incorporated into low-fat hard cheese. *Food Chemistry*.

[32] Ghorbanzade T., Jafari S. M., Akhavan S., Hadavi R. (2017). Nano-encapsulation of fish oil in nano-liposomes and its application in fortification of yogurt. *Food Chemistry*.

[33] Feller S. E., Gawrisch K., MacKerell A. D. (2002). Polyunsaturated fatty acids in lipid bilayers: Intrinsic and environmental contributions to their unique physical properties. *Journal of the American Chemical Society*.

[34] Rasti B., Jinap S., Mozafari M. R., Yazid A. M. (2012). Comparative study of the oxidative and physical stability of liposomal and nanoliposomal polyunsaturated fatty acids prepared with conventional and Mozafari methods. *Food Chemistry*.

[35] Hasan M., Belhaj N., Benachour H. (2014). Liposome encapsulation of curcumin: Physico-chemical characterizations and effects on MCF7 cancer cell proliferation. *International Journal of Pharmaceutics*.

[36] Huh N.-W., Porter N. A., McIntosh T. J., Simon S. A. (1996). The interaction of polyphenols with bilayers: Conditions for increasing bilayer adhesion. *Biophysical Journal*.

[37] Colas J.-C., Shi W., Rao V. S. N. M., Omri A., Mozafari M. R., Singh H. (2007). Microscopical investigations of nisin-loaded nanoliposomes prepared by Mozafari method and their bacterial targeting. *Micron*.

[38] Toniazzo T., Berbel I. F., Cho S., Fávaro-Trindade C. S., Moraes I. C. F., Pinho S. C. (2014). *β*-carotene-loaded liposome dispersions stabilized with xanthan and guar gums: Physico-chemical stability and feasibility of application in yogurt. *LWT- Food Science and Technology*.

[39] Kumar A. B., Habbu P., Thimmasetty, Lakshman, Hullatti P., Kumar S R. (2016). Phytosomes as novel drug delivery system for herbal medicine - A review. *Systematic Reviews in Pharmacy*.

[40] Keawchaoon L., Yoksan R. (2011). Preparation, characterization and in vitro release study of carvacrol-loaded chitosan nanoparticles. *Colloids and Surfaces B: Biointerfaces*.

